# Prenatal genetic diagnosis of disseminated infantile myofibromatosis: a case report and literature review

**DOI:** 10.1186/s12920-023-01612-w

**Published:** 2023-08-11

**Authors:** Yan Lü, Yulin Jiang, Huanwen Wu, Qingwei Qi, Xiya Zhou, Qi Guo, Na Hao, Juntao Liu, Hua Meng

**Affiliations:** 1 Department of Obstetrics and Gynecology, National Clinical Research Center for Obstetric & Gynecologic Diseases, Peking Union Medical College Hospital, Chinese Academy of Medical Science & Peking Union Medical College, Beijing, China; 2 Department of Pathology, Peking Union Medical College Hospital, Chinese Academy of Medical Science & Peking Union Medical College, Beijing, China; 3 Department of Ultrasound, Peking Union Medical College Hospital, Chinese Academy of Medical Science & Peking Union Medical College, Beijing, China

**Keywords:** Infantile myofibromatosis, Prenatal diagnosis, *PDGFRB*, Genetic testing, Case report

## Abstract

**Background:**

Infantile myofibromatosis (IM) is a rare disorder characterized by the formation of nodules in the skin, muscle, bone, and, more rarely, visceral organs. Very few cases are detected prenatally, and the final diagnosis cannot be made until pathology is completed after birth. Here, we present a case of disseminated form IM (DFIM) with a diagnosis established on prenatal genetic grounds.

**Case presentation:**

A woman at 23 weeks of gestation was referred for ultrasound evaluation of fetal kidney abnormality. Generalized masses in the skin and muscle of the fetus developed at 28 weeks. Prenatal genetic testing identified the pathogenic heterozygous variant c.1681C > T (p.R561C) of the *PDGFRB* gene inherited from the asymptomatic father. Intrauterine demise occurred at 31 weeks. Autopsy confirmed DFIM with involvement of the heart and kidney. All cases of prenatally detected IM were reviewed, revealing an association of high mortality with DFIM.

**Conclusions:**

Prenatal IM diagnosis is difficult. Initial detection is always based on ultrasound. DFIM has high mortality. The germline p.R561C mutation in *PDGFRB* may cause fetal demise due to severe visceral involvement of IM. Prenatal genetic testing provides a diagnosis before pathological results are available, leading to better counseling and management of pregnancy with a fetus with IM.

**Supplementary Information:**

The online version contains supplementary material available at 10.1186/s12920-023-01612-w.

## Background

Infantile myofibromatosis (IM) is a mesenchymal disorder characterized by the formation of nodules in the skin, muscle, bone, and, more rarely, visceral organs. Although rare, IM is the most common fibrous tumor of infancy and early childhood, with an incidence of 1:150,000 [1]. Mutations in the *PDGFRB* and *NOTCH3* genes have been identified as a cause of IM and are transmitted in an autosomal dominant mode [2, 3]. IM can be divided into the solitary form (SFIM), multicentric form without visceral involvement (MFIM), or disseminated form with visceral involvement (DFIM) [4]. The prognoses of SFIM and MFIM are usually good, while the mortality rate of DFIM is up to 73% [5]. Although spontaneous tumoral regression is typical, progression or recurrence is not rare; therefore, management should be individualized. In cases of SFIM or MFIM, therapeutic abstention and patient observation may be reasonable, while in cases of DFIM, surgical resection, chemotherapy, radiotherapy, and targeted therapy may be indicated [6].

Prenatal diagnosis is important since the parent can better plan for postnatal management or opt to terminate the pregnancy in severe cases. Very few cases are detected prenatally [4, 7–20]. In these cases, although tumors were detected by ultrasound (US), the final diagnosis was not made until pathology results were obtained after birth. In the present study, we present a case of DFIM. The diagnosis was established on prenatal genetic grounds.

## Case presentation

A 35-year-old Chinese woman, gravida 4 para 0, was referred to our hospital for unilateral enlarged fetal kidney at 23 weeks of gestation. Her three previous pregnancies were all biochemical pregnancies. Her past medical and family history was otherwise uneventful. She denied consanguineous marriage to her 35-year-old husband or any exposure to teratogens.

**Fig. 1:**
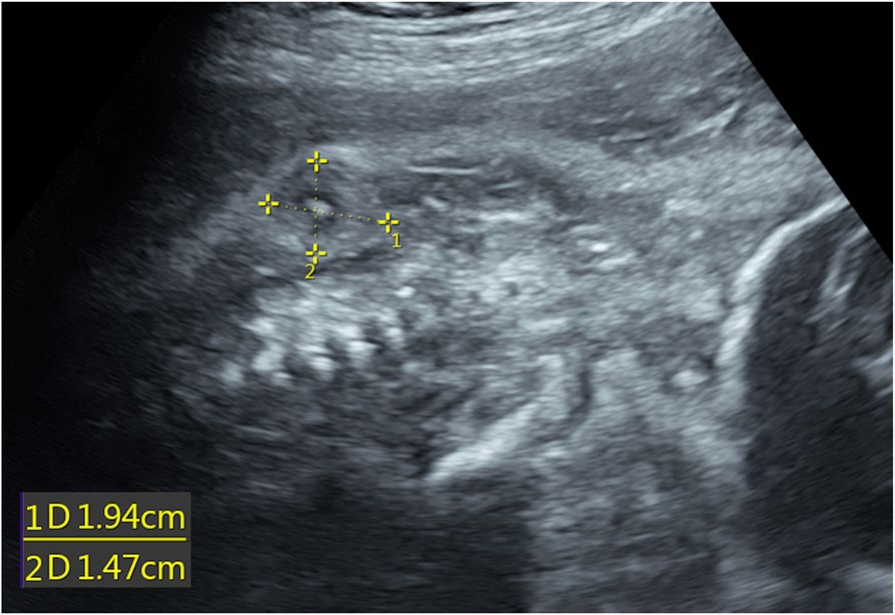
The longitudinal view of the fetus shows a paraspinal mass below the right scapula (calipers). Within the moderately echogenic mass, a hypoechogenic rim surrounds the hyperechogenic center

Prenatal healthcare of the present pregnancy was initially undertaken at a local hospital, and surveillance before 23 weeks was uneventful. An enlarged hyperechogenic fetal kidney on the left side was confirmed at our hospital at 26 weeks. The fetus was closely monitored with serial US examinations. Multiple avascular masses with well-defined margins in the skin and muscle of the forehead, chest, abdomen, and paraspinal region, with a maximum size of 1.9 $\times$ 1.5 mm, were found at 28 weeks (Fig. 1). The masses were hypoechogenic or moderately echogenic, and some were anechogenic or hyperechogenic inside. Other new findings were hyperechogenic fetal bowel and mild ascites. The differential diagnosis included soft tissue tumors such as myofibromatosis, neurofibromatosis, and hemangioma.

The parent was provided with pretest counseling, and amniocentesis was performed to detect the potential cause of the fetal ultrasound abnormality. Aneuploidy and copy number variations (CNVs) were excluded by low-pass CNV sequencing, which yielded negative results. Trio whole-exome sequencing (WES) was performed simultaneously. The extracted DNA was captured by an Agilent SureSelectXT Human All Exon V6 capture kit (Agilent Technologies, Santa Clara, CA, USA) and sequenced on a NovaSeq 6000 (Illumina, San Diego, CA) platform with 150-bp paired-end sequencing. A heterozygous variant, namely, c.1681C > T, in exon 12 of the *PDGFRB* gene (MIM 173410; RefSeq accession Number NM_002609.4) was inherited from the father (Fig. S1). Sanger sequencing of the variant c.1681C > T in *PDGFRB* was consistent with the exome sequencing, indicating that the proband and the father both carried this variant, but the mother’s gene was normal (Fig. S2). It was a missense mutation leading to the amnio acid substitution p.Arg561Cys. The variant is classified as pathogenic according to the American College of Medical Genetics and Genomics and the Association for Molecular Pathology (PS2_VeryStrong, PS3, PS4, PP1_Strong, PM2_Supporting, PP3) [21]. This variant was also predicted to be pathogenic by the Varsome (http://varsome.com) search engine with 13 points, including PP5_Very Strong (8pts), PM1_Moderate (2pts), PP3_Moderate (2pts) and PM2_Supporting (1pts). Further collection of the father’s medical history revealed a solitary skin nodule that regressed spontaneously. Of note, his mother, his uncle and he all had a skin nodule.

US examination at 30 weeks showed that the fetal masses increased in size, along with bilateral enlarged hyperechogenic kidneys and an increased ratio of cardiac to thoracic circumference and oligohydramnios. The parents were consulted and decided to terminate the pregnancy. However, fetal hydrops with pleural effusion and skin edema developed quickly and resulted in fetal demise at 31 weeks. Labor was induced, and a male fetus weighing 2010 g was delivered vaginally.

**Fig. 2:**
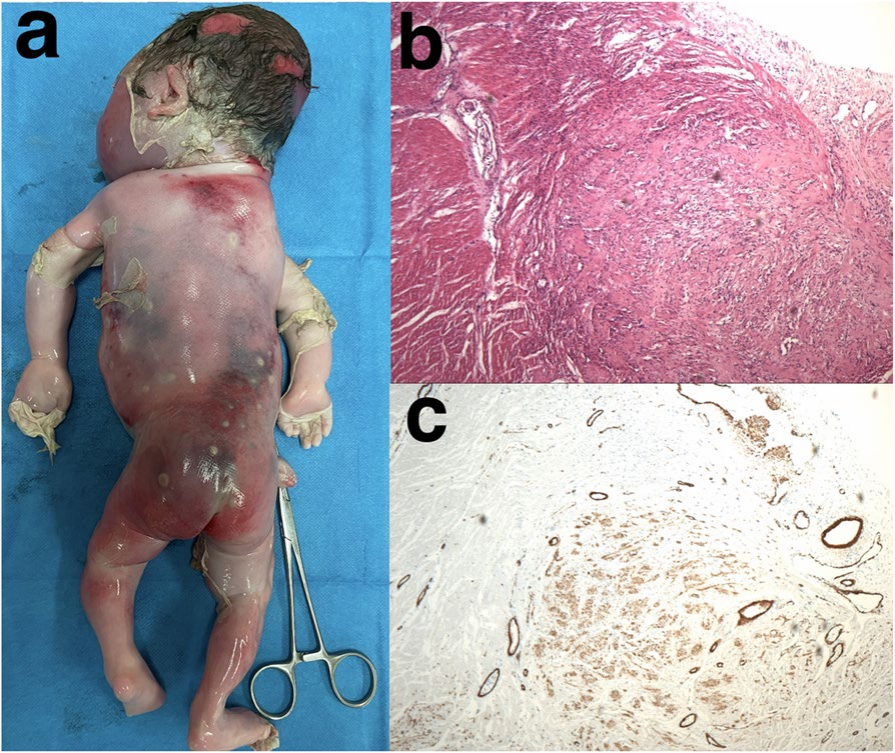
Macroscopy and microscopy of the fetus with disseminated infantile myofibromatosis. **a** Generalized masses located on the fetal back and extremities. **b** Hyperplastic nodules of spindle-shaped cells with eosinophilic cytoplasm of the fetal heart (H&E staining $\times 4$). **c** Immunopositivity of smooth muscle actin in myofibroblastic cells (SMA $\times 4$)

On autopsy, generalized masses in the skin and muscle of the fetal head, neck, trunk, extremities, and retroperitoneal soft tissue were noted (Fig. 2a). These mixed solid and cystic masses were ovoid, measuring from 1 to 3 cm. Microscopic examination of the masses showed multifocal hyperplastic nodules of spindle-shaped cells. Hyaline degeneration and calcification were encountered (Fig. 2b). Immunohistological staining demonstrated positivity for smooth muscle actin (Fig. 2c). Visceral involvement was confirmed in the heart and kidneys. The pathological results were consistent with the diagnosis of DFIM.

The parent was provided with genetic counseling for future pregnancy. Since the fetus inherited the mutation from the father in an autosomal dominant mode, the risk of IM in each of the offspring was 50%. Preimplantation or prenatal genetic diagnosis could be considered in the next pregnancy.

We identified 16 cases of prenatally detected IM through a literature review. The clinical characteristics and outcomes of all the published cases, as well as our case, are summarized in Table 1 [4, 7–20]. Most cases (15/17, 88%) were detected by US in the third trimester at a mean gestational age of 32 weeks, ranging from 13 to 38 weeks. SFIM was the most common type (8/17, 47%), followed by DFIM (6/17, 35%) and MFIM (3/17, 18%). Visceral involvement accounted for more than half of the cases (9/17, 53%). The involved visceral organs included the lung (3), liver (3), heart (2), spleen (1), intestine (1), and kidney (1). Ten patients (59%) underwent surgery after birth. Three patients (18%) underwent chemotherapy alone or in combination with surgery or imatinib. Twelve patients (71%) recovered well at the time of the last follow-up. Three patients (18%) died within one month after birth due to severe visceral involvement. One pregnancy with a large fetal paraspinal tumor was terminated at the request of the parent. Although the overall fetal mortality rate was 29% (5/17), it increased to 67% (4/6) for cases with DFIM.

**Table 1:** Published case reports of prenatally detected infantile myofibromatosis

First author (year)	Gestational week of detection	Type	Outcome
Nishioka et al. [7] (1999)	37	SFIM	Resection of chest wall tumor 7 days postdelivery
			No recurrence at 11 months of follow-up
Kubota et al. [8] (1999)	36	SFIM	Resection of left arm tumor 2 months postdelivery
			No recurrence at 3 years of follow-up
Meizner et al. [9] (2000)	30	DFIM	Paraspinal region and liver involvement
			Termination of pregnancy at 32 weeks
Muraoka et al. [10] (2008)	32	SFIM	Splenectomy 20 days postdelivery
			No recurrence at 3 years of follow-up
Arabin et al. [11] (2009)	13	DFIM	Resection of superficial head tumor, inoperable for intestinal involvement
			Died from sepsis 12 days postdelivery
Yeniel et al. [12] (2013)	32	SFIM	Resection of lung tumor 2 days postdelivery
			No recurrence at 1 year of follow-up
Zhang et al. [13] (2014)	38	SFIM	Resection of back tumor 3 months postdelivery
			No recurrence at 2 years of follow-up
Ushida et al. [14] (2017)	24	SFIM	Large mediastinal tumor breaking through the diaphragm and invading the liver
			Died from cardiac-respiratory failure and disseminated intravascular coagulation 5 days postdelivery
Pekar-Zlotin et al. [15] (2019)	34	DFIM	Generalized tumors located on the fetal head, neck, trunk, and lower extremity with heart involvement
			Died from cardiac failure 30 days postdelivery
Rekawek et al. [16] (2019)	36	MFIM	Generalized tumors located on the fetal head, trunk, and extremities
			No recommendation for surgery, asymptomatic at 1 year of follow-up
Fraissenon et al. [4] (2020)	32	SFIM	Resection of right flank tumor
Fraissenon et al. [4] (2020)	35	DFIM	Generalized tumors located on the fetal trunk and extremities with lung involvement
			Responded well to chemotherapy
Wang et al. [17] (2020)	34	MFIM	Multiple tumors located on the fetal lip, iliopsoas, and lower extremity
			Resection of larger facial tumor 2 weeks postdelivery, followed by chemotherapy
			No recurrence at 3 years of follow-up
De Martino et al. [18] (2021)	33	MFIM	Multiple tumors located beneath the fetal skull and lower extremity
			Resection of skull-based tumor
			Lower extremity tumors shrank at 2 years of follow-up
Popa et al. [19] (2021)	30	SFIM	Resection of left thigh tumor 2 months postdelivery
			No recurrence at 1 year of follow-up
Proust et al. [20] (2021)	34	DFIM	Multiple tumors located on the fetal back and lower extremity with liver and lung involvement
			Complete remission after imatinib and chemotherapy
			No recurrence at 3 years of follow-up
Our case	28	DFIM	Generalized tumors located on the fetal head, neck, truck, extremities, and retroperitoneal region with heart and kidney involvement
			Intrauterine demise at 32 weeks

## Discussion

Here, we describe the first case of DFIM diagnosed on the basis of prenatal genetic testing. Initial prenatal detection is always based on US imaging features, including (1) hypoechogenic or moderately echogenic homogeneity or slight heterogeneity, (2) clear demarcation, and (3) absence of or poor vascularity [4]. US is helpful in differentiating the diagnosis from other soft tissue tumors, such as highly hypervascularized hemangioma and frequently heterogeneous fibrosarcoma [4]. SFIM accounted for nearly half of the prenatally detected cases. However, the type of IM should be re-evaluated with careful examination after birth to determine whether IM is solitary or whether visceral organs are involved because small lesions may be neglected by US.

Although US provides important clues, the diagnosis of IM should always be ascertained on a pathological or genetic basis. The histopathological features of IM present as interlacing fascicles, nodules, or whorls of spindled myoid cells dispersed in a myxoid and collagenous stroma [1]. Immunohistological staining is positive for smooth muscle actin, vimentin, and sometimes CD34 [1]. Since biopsy is not available during pregnancy, the diagnosis of previously reported cases was based on pathology after birth.

We report the first case of IM diagnosed by prenatal genetic testing and confirmed by pathology postnatally. Germline or somatic heterozygous mutations in *PDGFRB* genes have been identified to account for IM [2, 20]. *PDGFRB* is located at 5q32 and encodes platelet-derived growth factor receptor beta (PDGFB), which is a cell surface tyrosine kinase receptor that plays an important role in embryogenesis and development [22] The gain-of-function c.1681C > T (p.R561C) mutation compromises autoinhibition by altering the binding of a juxtamembrane region to the catalytic site, so the receptor is activated in the absence of its ligand, leading to constitutive kinase firing and the formation of myofibromas in tissues with high *PDGFRB* signaling activity [2, 23, 24].

The p.R561C mutation is a recurrent germline mutation causing dominant inheritance in the familial form of IM with incomplete penetrance and variable expressivity [2, 3]. The mutation can present in any type of IM [2]. The clinical type and outcome of all the published cases involving the p.R561C mutation are summarized in Table 2 [2, 3, 24, 25]. The germline c.1681C > T (p.R561C) and the somatic c.1998C > A (p.N666K) mutation in *PDGFRB* were identified in one case (Family 5 in Table 2) [24]. It was proposed that the germline mutant triggered the development of myofibromatosis when combined with a second hit, possibly a somatic one [23, 24]. However, this phenomenon was not observed in other reported cases in Table 2. The average sequencing coverage of trio-WES in our case was 100X, and no other mutation was found. Therefore, the second-hit hypothesis still needs solid evidence and further validation. We reported the first case of fetal demise due to severe visceral involvement of myofibromatosis. Kidney abnormalities occurred prior to generalized subcutaneous lesions. Severe visceral involvement, together with heart lesions later proven by autopsy, was thought to be causative of rapidly progressing fetal hydrops and intrauterine demise. Our cases broaden the prenatal phenotype of IM and suggest that the p.R561C mutation could be strong enough to fully activate *PDGFRB* and cause fetal demise due to incompatibility with fetal growth.

**Table 2:** Published infantile myofibromatosis case reports involving c.1681C > T (p.R561C) mutation in *PDGFRB*

First author (year)	Familia cases (number of people affected)	Type	Outcome
Cheung et al. [2] (2013)	Family 1 (3)	SFIM	No need for further treatment
		MFIM	The majority regressed spontaneously
		DFIM	Treated
	Family 2 (2)	MFIM	NA
		NA^a^	NA
	Family 3 (3)	NA^a^	Resolved spontaneously
		MFIM	Resolved spontaneously at the age of 4 years
		MFIM	Resolved spontaneously at the age of 4 years
	Family 4 (3)	SFIM	Resected
		DFIM	NA
		MFIM	Resolved spontaneously
Art et al. [24] (2017)	Family 5 (1)	DFIM	Resection of the skull lesion and other resolved spontaneously
Martignetti et al. [3] (2013)	Family 6–12 (40)	NA	NA
Mudry et al. [25] (2017)	Family 7 (3)	DFIM	Partial recovery after chemotherapy and target therapy
		NA	Partial recovery after chemotherapy and target therapy
		NA	NA

^a^The patient had multiple myofibromas, but whether the visceral organ was involved was not documented

## Conclusions

Prenatal diagnosis of IM is difficult. Initial detection is always based on US. Prenatal genetic testing provides a solid diagnosis before pathological results are available, leading to better counseling and management of pregnancy with a fetus having IM. All prenatally detected cases are summarized to improve the understanding of this rare disease and reveal the association of poor prognosis with DFIM. The germline p.R561C mutation in *PDGFRB* may cause fetal demise due to severe visceral involvement of IM.

### Supplementary Information

**Additional file 1:** **Fig. S1.** Whole-exome sequencing identified the heterozygous variant c.1681C>T in the *PDGFRB *geneinherited from the father. The HGVS nomenclature of the variant is NC_000005.9:g.149505134G>A (GRCh37). The variant is indicated in a red box in the integrative genomics view.

**Additional file 2:** **Fig. S2.** Sanger sequencing of the variant c.1681C>T in *PDGFRB* was consistent with the exome sequencing, indicating that the proband and the father both carried this variant, but the mother’s gene was normal.

## Data Availability

The datasets used and/or analyzed during the current study are available from the corresponding author on reasonable request.

## Abbreviations

CNVs: Copy number variations
DFIM: Disseminated form infantile myofibromatosis
IM: Infantile myofibromatosis
MFIM: Multicentric form infantile myofibromatosis
PDGFRB: Platelet-derived growth factor receptor beta
SFIM: Solitary form infantile myofibromatosis
US: Ultrasound
