# Supplementary figures and images for: Prenatal genetic diagnosis of disseminated infantile myofibromatosis: a case report and literature review
Source: BMC Med Genomics. 2023 Aug 11;16:185. doi: 10.1186/s12920-023-01612-w (PMC10416477; doi:10.1186/s12920-023-01612-w)

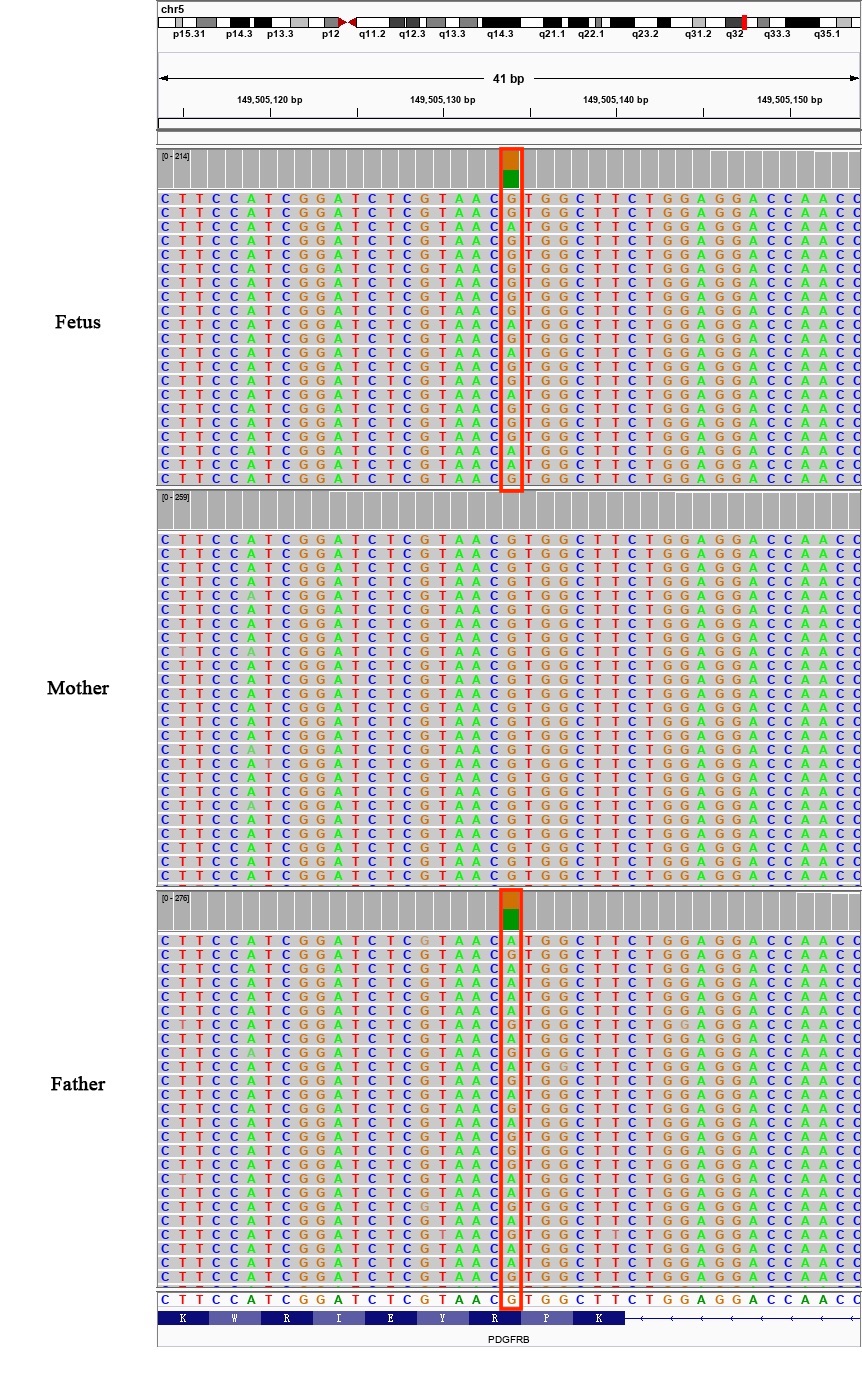

Supplement: Supplementary file 1 — Additional file 1: Fig. S1. Whole-exome sequencing identified the heterozygous variant c.1681C>T in the PDGFRB geneinherited from the father. The HGVS nomenclature of the variant is NC_000005.9:g.149505134G>A (GRCh37). The variant is indicated in a red box in the integrative genomics view. [file 12920_2023_1612_MOESM1_ESM.jpg]

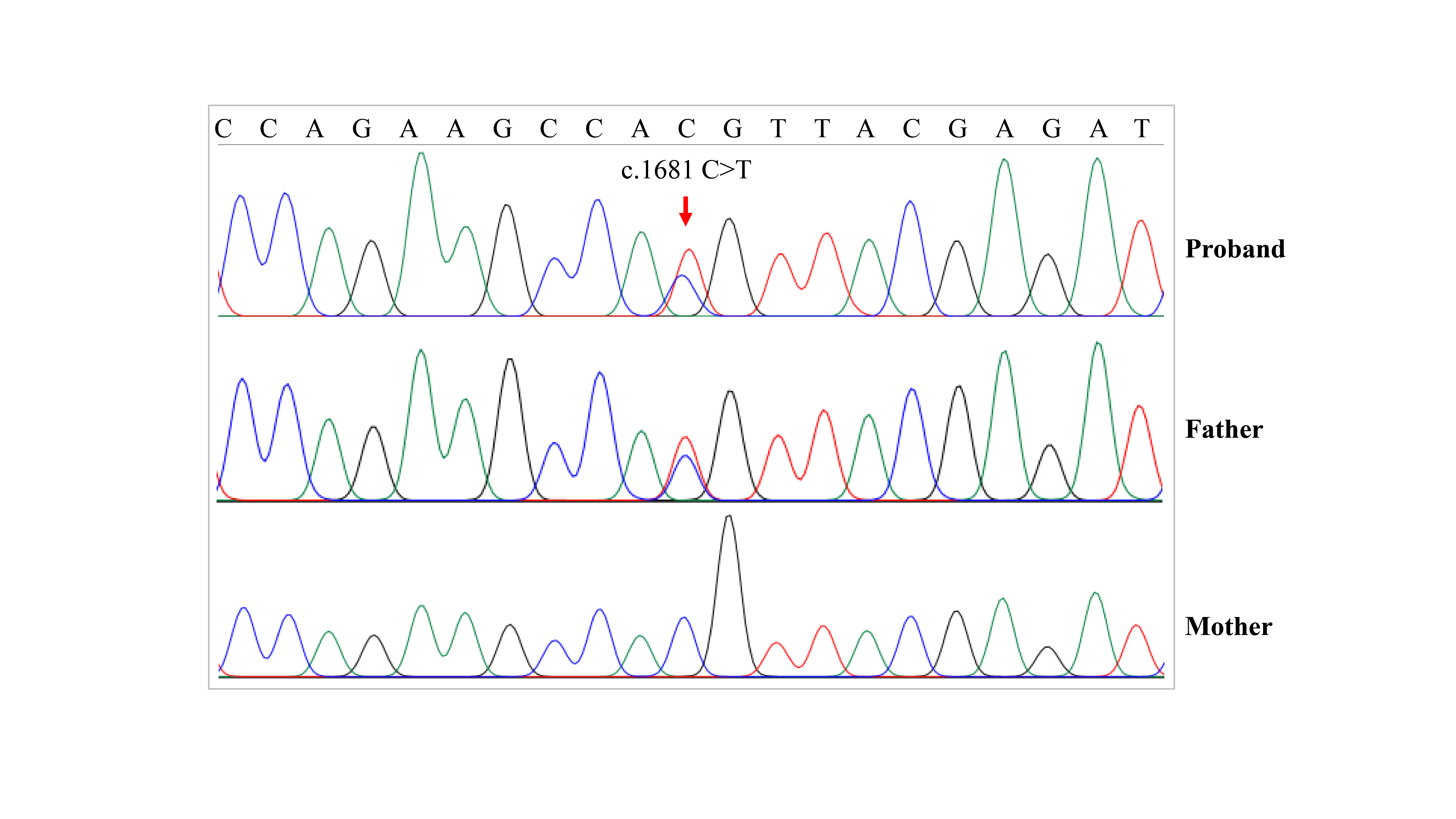

Supplement: Supplementary file 2 — Additional file 2: Fig. S2. Sanger sequencing of the variant c.1681C>T in PDGFRB was consistent with the exome sequencing, indicating that the proband and the father both carried this variant, but the mother’s gene was normal. [file 12920_2023_1612_MOESM2_ESM.tif]
